# From Molecular to Radionuclide and Pharmacological Aspects in Transthyretin Cardiac Amyloidosis

**DOI:** 10.3390/ijms26010146

**Published:** 2024-12-27

**Authors:** Silviu Marcel Stanciu, Ruxandra Jurcut, Ruxandra Dragoi Galrinho, Constantin Stefani, Daniela Miricescu, Ioana Ruxandra Rusu, Georgiana Sabina Prisacariu, Raluca Mititelu

**Affiliations:** 1 Department of Internal Medicine and Gastroenterology, Carol Davila University of Medicine and Pharmacy, Central Military Emergency University Hospital, 010825 Bucharest, Romania; silviu.stanciu@umfcd.ro; 2 Department of Cardiology, Carol Davila University of Medicine and Pharmacy, Institute of Cardiovascular Diseases “Prof CC Iliescu”, 022322 Bucharest, Romania; ruxandra.jurcut@umfcd.ro; 3 Department of Cardiology and Cardiovascular Surgery, University and Emergency Hospital, 050098 Bucharest, Romania; 4 Department I of Family Medicine and Clinical Base, “Dr. Carol Davila” Central Military Emergency University Hospital, 010825 Bucharest, Romania; constantin.stefani@umfcd.ro; 5 Discipline of Biochemistry, Faculty of Dentistry, Carol Davila University of Medicine and Pharmacy, 050474 Bucharest, Romania; 6 Discipline of Anatomy, Carol Davila University of Medicine and Pharmacy, 050474 Bucharest, Romania; ioana.rusu@umfcd.ro; 7 Clinic of Nuclear Medicine Central University Emergency Military Hospital “Dr Carol Davila”, 10825 Bucharest, Romania; georgiana.pscr@gmail.com (G.S.P.); raluca.mititelu@umfcd.ro (R.M.); 8 Department of Nuclear Medicine, University of Medicine and Pharmacy Carol Davila, 030147 Bucharest, Romania

**Keywords:** protein misfolding, cardiac amyloidosis, transthyretin cardiac amyloidosis, TTR knockdown, TTR stabilizers, TTR depleters, radionuclide imaging

## Abstract

Amyloidosis is a rare pathology characterized by protein deposits in various organs and tissues. Cardiac amyloidosis (CA) can be caused by various protein deposits, but transthyretin amyloidosis (ATTR) and immunoglobulin light chain (AL) are the most frequent pathologies. Protein misfolding can be induced by several factors such as oxidative stress, genetic mutations, aging, chronic inflammation, and neoplastic disorders. In ATTR cardiomyopathy (ATTR-CM), the amyloid fibrils can be found in the myocardium interstitial space and are associated with arrhythmias and heart failure. In pathological situations, the transthyretin (TTR) configuration is destroyed by proteolytic action, leading to monomers that further misfold and aggregate to form the amyloid fibrils. ^99m^Tc-Pyrophosphate (^99m^-Tc-PYP), ^99m^Tc 3,3-diphosphono-1,2-propanodicarboxylic acid (^99m^-Tc-DPD) and ^99m^-Tc hydroxy-methylene-Dyphosphonate (^99m^-Tc-HMDP) are used to detect myocardium amyloid deposits due to their ability to detect calcium ions that are present in the amyloid fibrils through dystrophic calcification. ATTR-CM therapy acts on different stages of the amyloidogenic process, including liver TTR synthesis, TTR tetramer destabilization, and misfolding of the monomers. The main aim of this narrative review is to present ATTR-CM, starting with molecular changes regarding the protein misfolding process and radionuclide aspects and finishing with pharmacological approaches.

## 1. Introduction

Amyloidosis is a rare and heterogeneous group of disorders [1] characterized by protein misfolding and protein deposits in various organs and tissues, leading to death in many cases [2,3]. Currently, it is considered that more than 50 disorders are associated with protein misfolding [4,5,6,7,8] and further associated with functional deficiencies and the generation of toxic intermediates [9,10].

During the past decade, the incidence and prevalence of cardiac amyloidosis (CA) have increased [11], especially among elderly persons [12]. CA was divided into two main subtypes, transthyretin cardiac amyloidosis (ATTR-CM) and immunoglobulin light chain cardiac amyloidosis (AL-CA), which are characterized by the specific pathogen protein [13,14].

Therefore, systemic amyloidosis with transthyretin (TTR) protein is the most common type of amyloidosis that induces cardiomyopathy [15], leading to heart failure (HF) and mortality worldwide [16].

Immunoglobulin light chain (AL) amyloidosis is characterized by the amyloid fibril deposits that can be found in the heart, gastrointestinal tract, kidneys, blood vessels, and peripheral nerves [17]. Moreover, in AL amyloidosis, the soluble light chains are converted into fibrillar aggregates that are associated with organ damage and dysfunction [18]. In AL amyloidosis, the toxic light chains are produced by a plasma cell clone or by a lymphoplasmacytic or marginal zone lymphoma in rare cases. These fibrillar deposits affect mostly the heart but also the kidneys [19] and are present in 50–75% of all AL patients [20]. In addition, the median survival rate for AL-CA patients with cardiac involvement is 6 months, while for those with ATTR-CM, this is between 26–43 months [21]. Transthyretin amyloidosis (ATTR) is characterized by the extracellular deposition of amyloid fibrils that contain the TTR protein [22], which suffers a conformational transformation, generating amyloid fibrils [23].

Amyloid cardiomyopathy is characterized by the extracellular deposition of amyloid fibrils induced by misfolding of the secreted light chains or TTR protein [24]. Partial or systemic ATTR is induced by misfolded TTR aggregation of a mutant or wild-type protein [25]. Regarding ATTR-CM, the amyloid fibrils can be found in the myocardium interstitial space, which is further associated with HF and arrhythmias [26].

## 2. Protein Misfolding and ATTR-CM

Proteins are one of the most important constituents of living cells [27,28]. They have three or four stages of organization [29] that induce them to form the biologically active form [30].

Molecular chaperons participate in the folding and assembly of the newly synthesized chain [31,32,33,34,35,36,37,38,39]. In healthy conditions, chaperones, together with the ubiquitin-proteasome pathway (UPP), the ubiquitin-proteasome system (UPS), and macro-autophagy, are involved in the degradation or refolding of the misfolded proteins [40,41,42,43,44,45,46,47].

**Figure 1:**
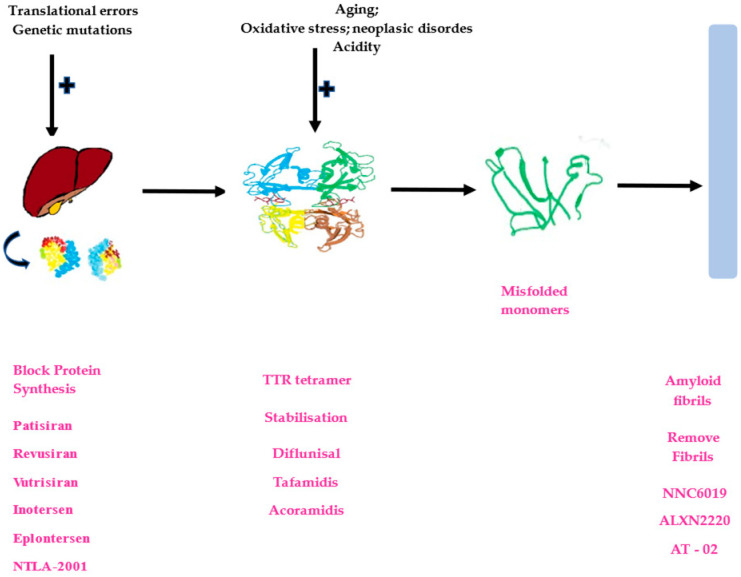
Transthyretin cardiac amyloidosis development. The development of this pathology can be induced by various factors. First of all, genetic mutations and translational errors lead to abnormal transthyretin protein synthesis. Further other factors such as aging, oxidative stress, neoplastic disorders, and acidity can destroy the normal configuration of TTR tetramer proteins with the formation of the misfolded monomers, leading to amyloid fibrils. Currently, several drugs have been developed with different actions, with some blocking hepatic transthyretin (TTR) synthesis (patisiran, revusinan, vutrisiran, inotersen, eplontersen, and NTLA-2001), others acting on TTR stabilization (difluninal, tafamidis, and acoramidis), and some trying to remove the amyloid fibrils (NNC6019, ALXN2220, and AT-02).

Protein misfolding can be induced by several factors such as genetic and somatic mutations, thermal and oxidative stress, local and non-local interactions, translational errors, metal ions, aging, inflammation, and neoplastic disorders and can take place inside or outside the cells [40,41,42,43,44,45,46,47,48,49,50,51,52,53,54,55,56,57] (Figure 1).

The term amyloidosis refers to the misfolding and/or partial unfolding of at least 27 different proteins [58] and can affect various organs such as the heart, eyes, kidneys, and central nervous system [59]. Although studies have shown that more than 30 proteins have been identified to cause amyloidosis, only two, AL and TTR, are the most important [60].

CA can be induced by various proteins, one of them being TTR, leading to ATTR-CM in 18% of cases. ATTR-CM can be divided into familial amyloid cardiomyopathy (FAC/hereditary ATTR-CM; HTTR-CM) and senile systemic amyloidosis (SSA/wild-type ATTR-CM; ATTR-CMwt) [61]. ATTR can be ATTRwt or mutant (variant ATTR, ATTRv), with systemic deposits of TTR molecules [62]. In ATTRwt amyloidosis, protein deposits are present in the heart but can also be found in bilateral carpal tunnel syndrome, spinal stenosis, and even biceps tendon rupture [63]. Moreover, in ATTRv, the extracellular deposition contains insoluble proteins [64]. Hereditary ATTR (HATTR) is a life-threatening autosomal dominant systemic amyloidosis induced by variant TTR mutation [22].

The *TTR* gene is located on chromosome 18 and encodes a protein [65] with four identical monomeric subunits, which is 55 kDa large and contains 127 amino acids with a β-sheet structure [66].

TTR has two anti-parallel β-strands notated from A to H, with one short α-helix structure between E and F, building a β-barrel structure. These two subunits form a dimer because they interact through hydrogen bonds formed by F and H β-strands. The other two subunits associate back-to-back by hydrophobic interactions realized by residues from β-strands A and B, and G and H form the tetramer structure [67].

TTR is a homotetrameric protein from plasma and cerebrospinal fluid, synthesized by the liver and the choroid plexus [68]. In pathological conditions, TTR protein aggregates and stores in tissue and various organs [69], such as nerves and the heart, inducing progressive and debilitating polyneuropathy and life-threatening cardiomyopathy [70,71,72,73,74].

The most important mutations of the TTR protein that are associated with cardiac pathology involve substitutions of valine (Val) with isoleucine (Ile), threonine (Thr) with alanine (Ala), leucine (Leu) with methionine (Met), and Ile with leucine (Leu) [75,76,77].

Furthermore, the replacement of the amino acid Val with Met at position 30 is associated with a late onset of disease but correlated with various symptoms, including cardiac ones [78]. ATTRv is induced by the substitution of Val from position 122 with Ile [79]. Worldwide, around 3–4% of African individuals have TTR protein mutations, including the Val to Ile substitution at position 122, which is associated with ATTRv-CM [80].

In addition, hereditary ATTRv-CM is associated with more than 110 *TTR* mutations [81], where severe progressive axonal polyneuropathy (ATTRv-PN) and cardiomyopathy (ATTRv-CM) are the most prominent manifestations [82]. Studies have reported that any polypeptide in certain conditions can form linear and rigid amyloid deposits [58,83,84,85,86,87,88,89,90,91]. Therefore, TTR suffers conformational changes associated with toxic extracellular deposits, found in the heart, nervous system, and other organs, leading to organ dysfunction [83,84,85,86,87,88,89,90,91].

## 3. From Molecular to Radionuclide Aspects in ATTR-CM

The realm of CA imaging has been greatly enriched by targeting various molecular markers through recent advancements in SPECT and positron emission tomography (PET) radiotracers. Among these, the most extensively researched are the ^99m^Tc-labeled bone-seeking tracers, such as diphosphonate and PYP compounds, known for their high sensitivity and specificity in diagnosing ATTR-CM. Additionally, ^18^F-sodium fluoride (NaF), a bone-avid PET tracer, and amyloid-specific PET tracers initially developed for Alzheimer’s disease, have shown promise in binding to both AL and ATTR deposits. A significant advantage of radionuclide imaging lies in its ability to perform whole-body scans concurrently, facilitating the evaluation of systemic multi-organ involvement. The differential uptake of radiotracers between ATTR and AL-CA remains under investigation, with hypotheses suggesting factors such as higher calcium content in ATTR and variations in myocardial fiber proteolysis influencing tracer affinity [92,93].

Radiopharmaceuticals labeled with ^99m^-Tc-^99m^Tc-PYP (^99m^Tc-Pyrophosphate), ^99m^Tc-DPD (^99m^Tc 3,3-Diphosphono-1,2-Propanodicarboxylic Acid), and ^99m^Tc-HMDP (^99m^Tc Hydroxymethylene Diphosphonate) have been demonstrated to be particularly effective in detecting amyloid deposits in the myocardium due to their affinity for calcium ions present in the amyloid fibrils through dystrophic calcification [94,95]. The presence of calcium in the damaged tissue is secondary to an enhanced ability of the denatured proteins of the injured cells to bind with calcium [96]. Nevertheless, the precise mechanisms responsible for the retrieval of the radiotracer from the bloodstream, passing via the endothelial cells, extracellular fluid, and ultimately reaching the calcium content of the amyloid deposits, remain unknown. When comparing endomyocardial biopsies of both ATTR-CM and AL-CA, it was determined that ATTR-CM presents with a greater density of microcalcification compared to AL-CA, thus accounting for the predilection of these tracers for ATTR-CM. In addition, it should be noted that not all bone-avid radiotracers labeled with ^99m^Tc exhibit a strong affinity for ATTR-CM. To date, ^99m^Tc-MDP (^99m^Tc Methylene Diphosphonate) is not recommended due to its limited sensitivity when compared to ^99m^Tc-PYP/DPD/HMDP [95].

Bisphosphonate scintigraphy to detect cardiac amyloidosis follows the same protocol as bone scans. Images are obtained 2–3 h after injection when using Tc-^99m^ DPD and HMDP scans, or after 1 h and 3h, respectively, when using Tc-^99m^PYP. The additional 3 h in the case of Tc-^99m^ PYP are required if significant blood pool activity is noted on the 1 h image [97].

While there may be some variation in local protocols for ^99m^Tc-labeled bone-avid radiotracer scans, it is generally standard practice to perform planar and SPECT (or SPECT/CT) imaging 2 to 3 h post-injection for ^99m^Tc-PYP/DPD/HMDP, and approximately 1 h (sometimes 3 h) post-injection for ^99m^Tc-PYP only [92]. Delayed imaging allows for clearance of activity in the blood pool, a process that may be prolonged in individuals with renal dysfunction or dilated cardiomyopathy. To ensure the reliability and accuracy of radionuclide procedure, standardized protocols should be followed at national and international levels [92,98].

Prior to tracer injection, the patient is informed about the purpose and expected results of the examination, as well as about how this examination is performed in this particular cardiac pathology (planar acquisitions, additional SPECT/CT). Relevant patient history may assist in interpreting final image results and includes a record of previous imaging exams, if available; any record of anatomical or functional abnormalities of the urinary system; and a history of medication that could influence the imaging results. If not contraindicated, good hydration is recommended in order to decrease blood pool activity and for radioprotection reasons. The quality of bone scintigraphy imaging results may be influenced by some medications that can alter tracer uptake patterns, potentially leading to artifacts. Agents such as aluminum, iron, and nephrotoxic chemotherapy may heighten renal tracer absorption, further burdening the renal system and affecting image quality. Similarly, androgen deprivation therapy used for prostate cancer treatment—such as estrogens and bicalutamide—can cause increased tracer uptake in the mammary tissue, particularly in cases of gynecomastia, potentially leading to interpretative challenges [99].

Planar and SPECT images are first assessed in order to detect diffuse myocardial uptake and differentiate myocardial uptake from activity in the blood pool. If myocardial uptake is identified, the physician proceeds to quantify it through the heart-to-contralateral lung ratio (H/CL), and/or by semi-quantitative comparison between uptake in the myocardium and ribs, awarding a score ranging from 0 to 3 [100,101]. Semiquantitative visual scoring (Perugini score) has a high diagnostic accuracy for ATTR-CM [101]. After the exclusion of AL-CA, translating myocardial uptake equal to grade 2 or greater than rib uptake (grade 3) has been associated with a specificity and positive predictive value of 100%. Despite its high sensitivity, grade 2/3 myocardial uptake is only ~70% specific in diagnosing CA [95,102]. Notably, patients with AL-CA may also present with grade 2 or 3 myocardial radiotracer uptake, outlining the importance of excluding AL-CA before diagnosing ATTR-CM using bone scintigraphy [100,103].

False positive results have been reported in cases of valvular/annular calcification or due to extracardiac uptake. Another potential pitfall in the interpretation of bone-avid scintigraphy in CA applies to acute myocardial infarction (MI), leading to false positive results if images are not postponed until a minimum of 4 weeks following an acute MI [100]. This is particularly important in older patients, who can have multiple pathologic associations, with some predisposing to ATTR-CM, i.e., aortic stenosis [104,105].

Recent findings suggest that using hybrid SPECT/CT systems over SPECT-only systems allows for attenuation correction and also provides enhancement of the myocardial boundaries [106]. Due to its ability to distinguish blood pool activity from myocardial uptake, SPECT should be performed in all patients referred for ^99m^Tc-PYP or DPD scintigraphy, rather than being limited to only those with an inconclusive planar examination [92,107].

Despite its high sensitivity for detecting potential ATTR-CA, radionuclide imaging using bone-avid radiotracers is not sufficient on its own to confirm or exclude the diagnosis of cardiac amyloidosis. Consequently, when combined with extensive biochemical tests, it can accurately diagnose ATTR-CM without the need for a biopsy [108,109]. The 2021 ESC diagnosis algorithm for identifying these two most common subtypes of CA (AL and ATTR-CA) follows four main scenarios, based on the scintigraphy examination using ^99m^-PYP, DPD, or HMDP, together with serum and urine assessments of monoclonal proteins and FLCs (free light chains). When scintigraphy images show a Perugini score of grade 2 or 3, ATTR-CM can be diagnosed. If the monoclonal protein assessment is negative, genetic testing should be employed to differentiate between ATTRv and ATTRwt forms. However, if the Perugini score uptake is grade 1, histological confirmation of amyloid deposits (that may be found in the extracardiac tissues) is required [109,110].

Amyloid typing based on histology tests is also required if the scintigraphy images showing uptake grade 2 or 3 are accompanied by at least one abnormal monoclonal protein test. A negative (≤1) Perugini score coupled with at least one abnormal blood or urine test should quickly prompt the ruling out of AL cardiac amyloidosis. In this manner, cardiac magnetic resonance imaging (CMR) can be used to outline cardiac involvement. However, if CMR findings are inconclusive or CMR imaging cannot be performed promptly, cardiac or extracardiac histologic evidence of amyloid deposits should avoid a delay in diagnosis, and consultation with a hematologist should be warranted. Finally, if both tests are negative, an alternative diagnosis should be considered, with the likelihood of ATTR and AL amyloidosis being low. However, as bone scintigraphy can give false-negative results for some ATTRv mutations and rare subtypes of CA, CMR followed by cardiac or extracardiac biopsy should be considered if suspicion persists [97,109].

Bone scintigraphy is becoming increasingly popular as a non-invasive family screening modality in hereditary transthyretin cardiac amyloidosis. According to the 2021 position statement of the European Society of Cardiology (ESC) Working Group on Myocardial and Pericardial Diseases on the diagnosis and treatment of amyloidosis, it is recommended that bone scintigraphy screening occurs every three years if any of the first-line exams (the yearly ECG, echocardiogram, blood tests, and biannual Holter monitoring) show abnormalities. ATTR carriers without cardiac involvement should begin routine evaluations a decade earlier than the age at which the youngest member of the family exhibited symptoms or the typical age of onset for the specific variant. While first-line diagnostic tests facilitate the diagnosis of ATTRv-CM, around 25% of patients presenting with subclinical ATTRv-CM do not rouse any suspicion based on initial evaluations, highlighting the importance of the three-year interval for bone scintigraphy [109,111]. Furthermore, due to its capacity for identifying early amyloid deposition, bone scintigraphy is considered to be one of the pillars of ATTR-CA screening algorithms outside of family screening. Recent studies have underscored the role of bone imaging (i.e., Tc-^99m^PYP imaging) in detecting asymptomatic carriers of hereditary transthyretin amyloidosis (ATTRv). Abnormalities of the H/CL ratio have been shown in 83% of asymptomatic carriers and 100% of symptomatic carriers, indicating that deposition in the myocardium may precede bone scintigraphy before any end-stage signs of non-ischemic cardiomyopathy. These findings further support the established role of bone scintigraphy as an adjunct to the CA screening armamentarium in high-risk populations, including asymptomatic gene holders, HFpEF patients, and elderly subjects [97].

Notably, bone scintigraphy findings have been linked to significant mortality and hospitalization rates, reinforcing the need for early detection and prompt management of CA, accounting for the role of the examination when evaluating prognosis [92,110,112]. Strengthening interdisciplinary awareness and utilizing advanced techniques such as artificial intelligence can further improve the accuracy and speed of diagnosis, especially in populations with more complex clinical backgrounds [110,113].

Bisphosphonate scintigraphy is a cost-effective imaging modality and has fewer availability issues than very expensive MRI or CT scanners with powerful cardiac evaluation capabilities. Due to its availability and relatively low cost compared to other imaging modalities, it is at least suitable for screening patients at risk for ATTR-CM, such as patients with aortic stenosis or patients with carpal tunnel syndrome, to name a few [113,114]. Further research would also be needed to assess the need for BS screening in a larger population, as suggested by the retrospective results of the recent work by Nebhwani et al. [110]. While the role of bone scintigraphy in the diagnosis of ATTR-CM is well-established, its role in monitoring disease progression is still controversial [115]. However, changes in myocardial bone tracer uptake after therapy may represent an early marker for response to treatment [116].

The latest developments in PET imaging have leveraged amyloid-binding radiotracers that are structurally similar to thioflavin-T, a benzothiazole dye known for its enhanced fluorescence upon binding to amyloid fibrils. Originally designed for Alzheimer’s disease, these tracers have been repurposed to successfully image cardiac amyloidosis. ^11^C-Pittsburgh compound B (^11^C-PiB) was one of the early agents in this category, but its short half-life led to restricted application to facilities equipped with a cyclotron. In response to these limitations, subsequent tracers with a longer half-life, such as ^18^F-Florbetapir, ^18^F-Florbetapen, and ^18^F-Flutemetamol, have been developed. While facilitating the direct detection of amyloid fibrils and allowing for the quantification of global and regional amyloid burdens of the heart, these radiotracers have demonstrated poor differentiation between AL-CA and ATTR-CM [93,94]. Additionally, ^18^F-sodium fluoride (^18^F-NaF)—a bone-seeking agent—was investigated for its ability to bind to amyloid fibrils using principles similar to SPECT bone-avid tracers but with the advantage of quantification. Despite its consistent detection of cardiovascular microcalcification, ^18^F-NaF PET/CT showed a low target-to-background ratio and unclear diagnostic relevance [93,117], in addition to higher costs and the limited availability of PET scan equipment (Table 1).

Patients with systemic amyloidosis, including AL and HATTR types, presented a higher uptake of ^11^C-PiB in the myocardium 15–25 min after injection, which is further linked with worse clinical outcomes, including death, heart transplantation, and acute decompensated heart failure [118]. Moreover, higher SUV values were found in AL-CA imaging compared to ATTR-CM, with 100% diagnostic accuracy for AL-CA [119] (Table 1). Dual-isotope imaging using both ^99m^Tc-PYP scintigraphy and ^11^C-PiB PET scans can complement each other in diagnosing both AL-CA and ATTR-CM. A positive PET exam combined with a negative ^99m^Tc-PYP scan indicates AL-CA and early-onset HATTR-CM, while the reverse pattern indicates wild-type and late-onset ATTR amyloidosis [120]. While there is some overlap in uptake values between AL and ATTR on amyloid-targeting PET imaging, a systematic review also found that AL generally exhibits higher uptake values compared to ATTR [114]. This finding indicates that bone scans and amyloid-targeting PET can be utilized as complementary imaging techniques, as bone scans demonstrate greater efficacy in detecting ATTR-CM. Despite its proven sensibility in diagnosing cardiac amyloidosis, ^11^C-PiB PET imaging is limited by the brief radioactive decay period of 20 min, requiring an onsite cyclotron for production [121].

^18^F-labeled PET radionuclides present a notable advantage over ^11^C-PiB tracers, with a half-life longer than 100 min and higher clinical applicability. Myocardial retention of ^18^F-florbetapir is elevated in patients with cardiac amyloidosis, notably within AL subgroups compared to ATTR subgroups [122]. Additionally, ^18^F-florbetaben PET imaging has proven effective in accurately diagnosing and differentiating cardiac amyloidosis from hypertensive heart disease, with myocardial uptake emerging as an independent determinant of myocardial dysfunction [121]. Delayed cardiac uptake of ^18^F-florbetaben may distinguish AL-CA from ATTR amyloidosis, evidenced by higher sustained mean SUV in AL patients [123]. Assessment of therapeutic response by amyloid-directed PET imaging has also been employed, with the amyloid burden on the PET correlating well with changes in performance status and serological markers post-treatment. Integrating fluoride PET imaging with MRIs has been shown to enhance diagnostic accuracy for ATTR amyloidosis [120]. However, while PET imaging effectively discriminates cardiac amyloidosis from controls, particularly through quantitative analysis, it appears less sensitive than the more established nuclear medicine techniques using ^99m^Tc-PYP or ^99m^Tc-DPD in diagnosing cardiac amyloidosis (Table 1). Although preliminary experience with this group of imaging agents has demonstrated high sensitivity, especially for early disease identification, they do not provide a clear distinction between AL-CA and ATTR-CM. Therefore, their routine use would require implementing a dual-isotope imaging technique that combines bone scintigraphy and amyloid-targeted PET imaging [106]. Although this approach may offer comprehensive phenotyping of CA, its potential as a cost-effective screening tool is restricted due to its high expense.

**Table 1:** MI (myocardial infarction); H/WB (heart/whole body ratio); ^1^ identical between the 1 h and 3 h protocols (adapted from [119,124,125]).

Tracer	Target and Original Application	CA Type	Advantage	Pitfalls and Limitations
^99m^Tc-PYP	Microcalcification (Bone scintigraphy)	ATTR>>AL	98% sensitivity 96% specificity for ATTR-CM ^1^ H/CL ratio ≥ 1.5: 97% sensitivity 100% specificity for ATTR-CM	False positives in cases of acute MI, valvular/annular calcification, or due to extracardiac uptake. Additional 3 h imaging may be required if blood pool activity is noted.
^99m^Tc-DPD	Microcalcification (Bone scintigraphy)	ATTR>>AL	H/WB ratio > 0.091: 92% sensitivity and 88% specificity	Same as ^99m^Tc-PYP.
^99m^Tc-HMDP	Microcalcification (Bone scintigraphy)	ATTR>>AL	Comparable to ^99m^Tc-DPD	Same as ^99m^Tc-PYP.
^11^C-PIB	Amyloid (Brain imaging in Alzheimer dementia)	AL>>ATTR	Detects both AL-CA and ATTR-CM, ability to detect early disease. Can complement ^99m^Tc-PYP scintigraphy	Short half-life (20 min) limits practicality, requiring onsite cyclotron for generation. Lack of large-sized studies to confirm efficacy.
^18^F- Florbetapir/ Florbetaben/Flutebetamol/NaF	Amyloid (Brain imaging in Alzheimer dementia)	AL>>ATTR	Can diagnose both AL-CA and ATTR-CM. Allows for early detection and aids therapy response assessment	Lack of large-sized studies to confirm efficacy.

## 4. From Molecular to Pharmacological Approaches in ATTR-CM

Until the late 1990s, therapeutic approaches for ATTR-CM were limited to symptomatic treatment and, in some specific cases, liver transplantation or combined liver–heart transplantation. Advances in the last years in the understanding of molecular mechanisms involved in TTR amyloid formation led to a new era of specific therapies. Management of ATTR-CM involves two main strategies: (1) treatment of cardiovascular symptoms and prevention of complications and (2) specific therapy, aiming to change the natural course of the disease.

General therapeutic approaches for cardiovascular symptoms and complications focus on the treatment of heart failure symptoms, atrial and ventricular arrhythmias, and conduction disturbances, prevention of thromboembolism, treatment of orthostatic hypotension, and management of severe aortic stenosis. However, the purpose of this review is beyond discussing the general approach, instead focusing on emerging target therapy.

Disease-modifying therapy acts on different stages of the amyloidogenic process: liver synthesis of TTR, destabilization of the TTR tetramer, and misfolding of the monomers, as well as aggregation and deposition of amyloid fibrils in organs. Consequently, taking into consideration the mechanism of action, three types of therapy exist at present:

1. TTR knockdown: gene-silencing therapy (synthetic oligonucleotides) and gene-editing therapy (CRISPR-Cas9),
2. TTR stabilizers, and
3. TTR depleters: monoclonal antibodies.

### 4.1. TTR Knockdown

Gene-silencing therapy, namely RNA-targeting therapy, blocks translation at the mRNA level.

Synthetic oligonucleotides are small, single- or double-stranded oligonucleotides chemically modified in several ways in order to increase resistance to nucleases and to improve their target affinity and bioavailability, with as few “off-target” effects as possible. They bind to RNA via Watson–Crick base pairing in a target-specific manner in order to modify protein expression [126]. According to their mechanism of action, two types of therapeutic oligonucleotides were studied as gene-silencing therapies for ATTR-CM: small interfering RNAs (siRNAs), such as patirisran, revusiran, and vutrisiran, and antisense oligonucleotides (ASOs), such as inotersen and eplontersen.

siRNAs are double-stranded RNA molecules ranging from 19 to 29 nucleotides and composed of an antisense or “guide” strand to target mRNA and a sense or “passenger” strand. The mechanism of action of siRNA agents implies the delivery of a chemically synthesized molecule of siRNA into the cytoplasm of the target cell and loading it into the RNA-induced silencing complex (RISC). After removing the “sense” strand, the remaining “antisense” single-stranded siRNA component guides and aligns the RISC complex on the target mRNA. Through the action of a catalytic RISC protein (Argonaute-2), mRNA is cleaved, resulting in the decreased production of TTR [127].

ASOs consist of a single strand ranging from 16 to 30 nucleotides that can target complementary RNA in order to modify protein expression, which results in a reduction of serum protein. They generally follow the ‘gapmer’ pattern: a DNA core (internal “gap”) surrounded by RNA-based chemically modified flanking regions, often consisting of 2′ O-methoxyethyl, which promotes target binding and is resistant to the endogenous ribosome nuclease (RNase) H enzyme. Then, DNA-based oligonucleotides bind in a sequence-specific manner to their target mRNA transcripts and form the DNA/RNA hybrid. The endogenous RNase H enzyme recognizes the DNA/RNA hybrid and cleaves the duplex structure, causing mRNA degradation and silencing target gene expression [128].

Patisiran is a siRNA agent encapsulated in a lipid nanoparticle in order to protect the RNA molecule from degradation by circulatory endo- and exonucleases and to increase targeted delivery to hepatocytes through apolipoprotein-E-mediated uptake by LDL receptors. Patisiran is the first siRNA drug developed for ATTR amyloidosis. In 2018, based on the results of the APOLLO–A phase 3 trial, both the European Medicines Agency (EMA) and the United States Food and Drug Administration (FDA) approved it for the treatment of ATTRv polyneuropathy. Most adverse events were mild or moderate, and no clinically significant changes were identified in hematological, liver, or renal function. Serum transthyretin levels decreased by 80% in the patisiran group. Moreover, in a subgroup of patients with evidence of cardiac amyloid involvement at baseline, patisiran was associated, after 18 months, with reduced NT-proBNP concentrations and mean left ventricle wall thickness, as well as increased left ventricle global longitudinal strain, with the greatest differential increase observed in the basal region [129]. In a retrospective cohort study of 20 patients with ATTRv-CM, patisiran was associated with a decrease in median heart/whole-body ratio on bone scintigraphy after 29 months, while no changes in serum cardiac biomarkers, NYHA class, or echocardiographic parameters were observed [130]. This may suggest an early response to treatment, which precedes other traditional changes. The APOLLO-B phase 3 trial, designed to assess patisiran efficacy in the treatment of ATTR-CM, enrolled 360 patients with ATTRwt-CM or ATTRv-CM and evidence of heart failure, but who were clinically stable. In this trial, administration of patisiran over a period of 12 months resulted in preserved functional capacity. Nevertheless, the composite endpoint of death from any cause, cardiovascular events, and changes from baseline in the distance covered on the 6 min walk test was not significant, possibly due to the short monitoring period of the study [131]. Importantly, in all patients in the patisiran arm, the Perugini grade decreased or demonstrated no change from baseline at 12 months when evaluated by bone scintigraphy, while in the placebo arm, there was no decrease in Perugini grade at follow-up [132]. Therefore, studies of longer duration are necessary to determine whether the reduction in serum TTR or the regression of myocardial tracer uptake induced by patisiran is associated with a decrease in mortality and cardiovascular events in patients with ATTR-CM.

Revusiran is another siRNA agent, conjugated to the N-acetylgalactosamine (GalNAc) ligand, that specifically binds with high affinity to the asialoglycoprotein receptor that is highly expressed by hepatocytes. The ENDEAVOUR phase 3 trial evaluated revusiran for the treatment of ATTRv-CM. The study’s sponsor prematurely discontinued dosing, after only 7 months, due to an observed mortality imbalance between treatment arms (12.9% of patients on revusiran and 3% on placebo), yet no clear causative mechanism could be identified. Most patients died because of heart failure, yet they had more advanced heart failure symptoms at baseline [133].

Vutrisiran is another siRNA agent conjugated to the GalNAc ligand that is safe and has a good tolerability profile. It has been approved for ATTRv polyneuropathy in view of the HELIOS-A trial results [134]. In a cohort of patients with cardiac involvement, evaluated by bone scintigraphy from the HELIOS-A trial, 96% were stable or improved by ≥1 Perugini grade after 18 months of treatment with vutrisiran. Moreover, among patients with Perugini grades ≥ 1 at baseline, half of them improved by ≥1 Perugini grade and 16% improved by ≥2 Perugini grades. This suggests that the regression of cardiac amyloid infiltrates may precede clinical improvement [135].

The HELIOS-B phase 3 trial, designed to evaluate the efficacy and safety of vutrisiran on the reduction of all-cause mortality and cardiovascular hospitalizations and urgent heart failure visits at 36 months, enrolled 655 patients with ATTRwt-CM or ATTRv-CM and heart failure NYHA class I–III. The trial met its primary endpoint, demonstrating a statistically significant reduction in the composite of all-cause mortality and recurrent cardiovascular events in both the overall population (HR 0.72, *p*-value 0.01) and in the monotherapy population (patients not receiving tafamidis at baseline; HR 0.67, *p*-value 0.02) [136].

Inotersen is a 2′ O-methoxyethyl-modified ASO and is the first ASO developed for ATTR amyloidosis. Inotersen enters the nucleus of the hepatocyte and selectively binds to the 3′-untranslated portion of the complementary mRNA TTR, and mediates its degradation through cleavage by RNase H, consequently leading to a reduction in serum TTR. In 2018, the EMA and FDA approved it for the treatment of ATTRv polyneuropathy, taking into consideration the results of the NEURO-TTR phase 3 trial [137].

Nevertheless, some safety concerns were identified, including severe thrombocytopenia (<25.000/mm^3^), glomerulonephritis, and an imbalance in deaths (five patients in the inotersen group and none in the placebo group), due to different causes: cachexia, intestinal perforation, HF progression, and most importantly, one case of intracranial hemorrhage in a patient with severe thrombocytopenia. Therefore, inotersen is contraindicated in patients with less than 100,000 platelets/mm^3^, and frequent platelet monitoring is mandatory during this treatment. The mechanism for thrombocytopenia seems to be immune-mediated, as all patients with severe thrombocytopenia had anti-platelet antibodies in their serum and platelet counts returned to baseline or near-baseline levels after discontinuation of inotersen and treatment with glucocorticoids. Likewise, inotersen is contraindicated in patients with eGFR < 45 mL/min/1.73 m^2^ or a urine protein to creatinine ratio ≥ 113 mg/mmol, and close monitoring of renal function is required during treatment. Moreover, in the cardiomyopathy subgroup, after 15 months, inotersen failed to demonstrate a significant improvement in cardiac structural and sensible functional parameters, such as global longitudinal strain [137].

Eplontersen is an ASO with a sequence identical to inotersen, although it is conjugated to a triantennary N-acetyl galactosamine (GalNAc3), which facilitates binding to the asialoglycoprotein receptor expressed by hepatocytes, thus determining a potent knockdown of TTR [138]. In a cohort of patients with ATTRv-CM from the NEURO-TTRansform trial, eplontersen significantly reduced myocardial bone tracer uptake, suggesting that bone scintigraphy may be used to monitor the efficacy of specific therapies [139]. CARDIO-TTRansform is a phase 3, double-blind, randomized study designed to evaluate the efficacy of eplontersen compared to placebo in approximately 1400 participants with ATTR-CM and heart failure NYHA class I–III receiving the available standard of care. The primary endpoint is a composite outcome of cardiovascular mortality and recurrent cardiovascular clinical events occurring up to week 140. The trial started in 2020, enrolled 1438 patients until present, and the estimated study completion date is November 2025.

Gene-editing therapy has dramatically changed biomedical research. This revolutionary technology can correct errors in the genome by adding or ablating genes. CRISPR-Cas9 (clustered regularly interspaced palindromic repeats and associated Cas9 endonuclease) is a gene-editing technology that contains two main components: a guide RNA to match a desired target gene and a Cas9 endonuclease that binds to the DNA and cuts both strands, allowing modifications to the genome [140]. NTLA-2001 is a CRISP-Cas9 transported by a lipid nanoparticle that facilitates delivery to hepatocytes and consists of a single guide RNA to target the TTR gene and a Cas9 endonuclease to knock down the targeted gene. A first phase I clinical trial designed to evaluate the safety and pharmacodynamics effects of NTLA-2001 in six patients with ATTRv-PN with or without cardiomyopathy showed that after 28 days, a single dose of NTLA-2001 led to a dose-dependent decrease in serum TTR ranging from 47% to 96% with only mild adverse events [141]. Another phase I clinical trial (NCT04601051) designed to evaluate the safety, tolerability, pharmacokinetics, and pharmacodynamics of NTLA-2001 in participants with ATTRv-CM or ATTRwt-CM is ongoing and the estimated study completion date is August 2026.

Importantly, in the context of low TTR levels induced by TTR knockdown therapy, and because TTR binds retinol-binding proteins involved in vitamin A transport, patients need to take supplements of vitamin A to ensure its adequate delivery to tissues.

### 4.2. TTR Stabilizers

TTR stabilizers aim to maintain the natural tetramer structure of TTR by inhibiting dissociation into monomers, consequently preventing their misfolding and the deposition of amyloid fibrils into tissues. The mechanism of action is either by binding to the thyroxine binding site (e.g., tafamidis and diflunisal) or by forming hydrogen bonds at the bottom of the thyroxine-binding pocket, thus mimicking the structure of the naturally protective T119M variant (e.g., acoramidis).

Tafamidis has been approved for both ATTRv-CM and ATTRwt-CM by the FDA since 2019 and by the EMA since 2020, at once-daily dosages of 61 mg (free acid) or tafamidis meglumine at 80 mg. Similar to other specific therapy agents, a higher dose of tafamidis was associated with a reduction in myocardial bone tracer uptake in patients with ATTR-CM [116,142,143].

However, one retrospective cohort study reported an increase in the heart/whole-body ratio of ATTRv-CM patients treated with the lower dose of 20 mg of tafamidis for the indication of polyneuropathy. This finding emphasizes not only the importance of using the higher and correct dose of tafamidis for ATTR-CM, as approved by the FDA and EMA, but also suggests that bone scintigraphy may be a valid tool for the assessment of response to disease-modifying therapies [130].

The ATTR-ACT trial showed that tafamidis was associated with lower all-cause mortality than the placebo (29.5% vs. 42.9%; hazard ratio of 0.70; 95% confidence interval (CI) 0.51 to 0.96) and a lower rate of cardiovascular-related hospitalizations, with a relative risk ratio of 0.68 (0.48 per year vs. 0.70 per year; 95% CI, 0.56 to 0.81). Importantly, the largest effect was achieved in patients with NYHA functional class I and II. Furthermore, tafamidis reduced the decline in functional capacity and improved quality of life, as compared with the placebo, after 30 months of treatment. Moreover, tafamidis has a favorable side effect profile, with only mild or moderate adverse events and less frequent permanent discontinuation than in the placebo group [144]. A post hoc analysis of the ATTR-ACT trial showed that tafamidis attenuated the decline of LV systolic and diastolic function over 30 months in patients with ATTR-CM [145].

Diflunisal is a non-steroidal anti-inflammatory drug and showed improvements in cardiac troponin I, global longitudinal strain, and overall survival in a few small, single-center, retrospective studies. However, it is associated with adverse events such as gastrointestinal bleeding, renal dysfunction, fluid retention, or the aggravation of heart failure [146,147,148]. Nevertheless, larger studies are necessary.

Acoramidis is a strong TTR stabilizer that binds to the TTR tetramer with a higher affinity than thyroxine and determines near-complete stabilization (more than 90%) at the dose of 800 mg twice daily in both ATTRv and ATTRwt patients [149]. In the ATTRibute-CM trial, acoramidis showed favorable effects with regard to death from any cause, cardiovascular-related hospitalization, NT-proBNP level, and 6 min walk distance after 30 months of treatment. Moreover, it has a good safety profile, with the adverse events being mostly mild and no more than moderate [150]. In November 2024, acoramidis was approved by the FDA for the treatment of ATTR-CM and received a positive opinion from the Committee for Medicinal Products for Human Use of the EMA in December 2024.

### 4.3. TTR Depleters

Tafamidis, currently the only drug for ATTR-CM approved by both the FDA and EMA, and acoramidis, which has been approved by the FDA, influence just the progression of the disease, with no effect on tissue amyloid fibril deposits. Moreover, patients with ATTR-CM have a significant delay between the onset of symptoms and diagnosis; therefore, the diagnosis is often made during an advanced phase of the disease. Consequently, there is an emergent need to develop new classes of drugs, which can remove the existing TTR amyloid from tissues through phagocytic immune cells. This is the rationale behind creating TTR depleters.

NNC6019 (PRX004) is an investigational humanized monoclonal antibody designed to deplete pathological amyloid in patients with ATTR, without affecting the normal tetrameric form of the protein. The phase 1 trial (NCT03336580) was terminated prematurely due to the COVID-19 pandemic and the authors reported the results in an oral presentation at the American Academy of Neurology Virtual Annual Meeting in 2021. Besides safety and good tolerability, NNC6019 showed improvement in cardiac function for all seven evaluable patients after 9 months. There is an ongoing phase 2 trial (NCT05442047) enrolling patients with ATTR-CM, who are randomly assigned to receive one of two doses of NNC6019, or a placebo, once every four weeks for one year. The trial’s primary outcome is to assess changes in the 6 min walk test and in blood levels of NT-proBNP. The estimated study’s completion date is in May 2025.

ALXN2220 (NI006) is a humanized, recombinant monoclonal IgG1 antibody that selectively binds to an epitope exposed on an abnormal TTR protein in ATTR-CM, without binding to physiologically folded TTR. In a phase 1 trial, the use of ALXN2220 was not associated with any drug-related serious adverse events. Moreover, ALXN2220 showed, over a period of 12 months, a decrease in cardiac tracer uptake on scintigraphy and extracellular volume on cardiac magnetic resonance imaging, along with cardiac marker levels [151]. The DepleTTR-CM is the first phase 3 trial, which started in January 2024 and is meant to assess the efficacy of ALXN2220 versus placebo in patients with ATTR-CM. The primary endpoint is a composite endpoint of all-cause mortality and total cardiovascular clinical events over the treatment period of up to 48 months. The estimated number of patients to be enrolled is 1000 and the estimated study completion date is November 2028.

AT-02 is another humanized, recombinant IgG1–peptide fusion that binds human cardiac amyloid in tissue sections with high specificity and intensity and inhibits fibril growth, therefore promoting clearance of cardiac amyloid deposits [152]. In 2021, a multicenter, international, phase 1 trial (NCT05521022) was started, designed to evaluate the safety, tolerability, and pharmacokinetics of AT-02 in healthy volunteers and in subjects with a confirmed diagnosis of ATTR-CM, AL, or other form of systemic amyloidosis. The end date of this study is expected to be in March 2025. In 2023, a phase 2 open-label extension study was started to evaluate the long-term (up to 112 weeks) safety, tolerability, and clinical activity of AT-02, and the estimated completion date is in March 2026 (Figure 1).

### 4.4. Combination Therapy

Combining different therapies could be rational, taking into account their different mechanisms of action. Hence, it is reasonable to believe that simultaneously targeting both TTR production, with either gene-silencing or gene-editing therapies, and TTR stabilization may have a complementary and synergistic effect and improve patient outcomes.

However, there is currently scarce data regarding dual therapy in ATTR. The percentage of patients from the APPOLO-B trial on dual therapy (patisiran and tafamidis) is quite low at 25% (91 patients), and the sub-group analyses did not show any benefits of double therapy [131]. In the HELIOS-B trial, 40% of patients were taking tafamidis at baseline and, in a pre-specified subgroup analysis, the composite of all-cause mortality and recurrent cardiovascular events was reduced by more than 20% in these patients (HR 0.79; 95% CI 0.51–1.21) [136].

Further results are expected to come at the end of 2025 from the CardioTTRANSFORM trial, where an even greater percentage of patients on dual therapy is expected. Regarding this large trial, we will find out if combination therapy provides additional clinical benefits in ATTR-CM.

## 5. Conclusions

In some pathological conditions, protein structures can suffer irreversible modifications, leading to organ and tissue deposits. In these situations, soluble proteins adopt a cross-β-sheet conformation, generating insoluble extracellular fibrils. The *TTR* gene mutation causes TTR protein destabilization, where a TTR tetramer structure is destroyed by proteolytic action and the monomers will aggregate and form amyloid fibrils.

Advances in radionuclide imaging have greatly enhanced the diagnostic capabilities for ATTR-CM, particularly with bone-seeking tracers, which demonstrate high specificity and sensitivity. This imaging approach allows whole-body assessments, providing insight into systemic involvement. Though effective in diagnosing ATTR-CM, these bone-avid tracers require complementary biochemical tests and a standardized imaging protocol for reliable results.

Emerging PET tracers, which were initially developed for Alzheimer’s disease, offer potential in cardiac amyloidosis imaging, allowing quantitative amyloid load assessments. Despite its diagnostic strengths, PET imaging’s cost and equipment requirements constrain its widespread clinical use. Thus, while radionuclide imaging provides critical diagnostic insights into ATTR-CM, integrating dual modalities and biochemical assays may enhance diagnostic accuracy and optimize clinical utility for managing cardiac amyloidosis.

In the last few years, due to the great progress made in discovering the molecular mechanisms of amyloidogenesis, new emerging therapies were developed in order to improve both the survival and quality of life of patients with ATTR-CM. These therapies act directly on different stages of the amyloidogenic cascade. Accordingly, therapeutic options that are currently under investigation for ATTR-CM comprise various drugs that inhibit the hepatic synthesis of TTR, stabilize the tetramer, or disrupt the amyloid fibrils. At present, the only approved pharmacological agent for ATTR-CM worldwide is tafamidis, a TTR tetramer stabilizer that inhibits the accumulation of amyloidogenic species in different tissues. Moreover, the FDA recently approved acoramidis, another TTR tetramer stabilizer, for ATTR-CM due to the remarkable results of the ATTRibute-CM trial.

Nevertheless, with the results of large-scale clinical trials expected over the next few years, gene-silencing and gene-editing therapy and TTR depleters may represent another option of pharmacological agents licensed for the treatment of ATTR-CM. However, despite early diagnosis and specific treatment, ATTR-CM remains a progressively debilitating disease associated with high mortality.
